# A New Tool for Measuring the Brutality of War

**DOI:** 10.1371/journal.pmed.0050249

**Published:** 2008-12-16

**Authors:** Egbert Sondorp

## Abstract

Egbert Sondorp discusses the public health uses and implications of a new tool, the Dirty War Index, that measures prohibited war outcomes inflicted on populations during conflict.

## The Importance of Visualising Data

Earlier this year, Hans Rosling, Professor of International Health at the Karolinska Institute, Sweden, delivered the 2008 International Health Lecture for the Academy of Medical Sciences in London, a lecture entitled “A fact-based world view”. Rosling co-founded the Gapminder project (http://www.gapminder.org/): animated software that makes a multitude of quantitative datasets visible and meaningful. It was fascinating in his lecture to see health and wealth data from 166 countries move through time, making some important messages visible to a broad audience. For instance, Rosling showed the major health improvements over the last couple of decades in most countries in the world, in comparison to a small set of countries where all health development seems to have totally stagnated. This stagnant set of countries consists of most African countries and a number of conflict-affected countries elsewhere, such as Afghanistan.

In theory at least, mortality rates during war should not need the kind of visual tools that Rosling uses to make an impact—they should speak for themselves. For example, a series of mortality surveys conducted by the International Rescue Committee in the Democratic Republic of the Congo found an excess of almost 4 million deaths between 1998–2004, all attributable to the ongoing conflict [1]. However, when reported mortality rates are high, they may be challenged by those who do not like the message. Comparable mortality surveys in Northern Uganda and Darfur were contested, which undermined their credibility. A similar fate befell one of the mortality surveys in Iraq, which estimated that 650,000 deaths in Iraq had occurred since the invasion in 2003 [2]. While this number made the survey headline news, it was also subject to a range of criticisms that undermined the report's main message, which was that many had died as a result of the invasion.

Despite their potential to clearly convey the brutality of war, mortality surveys have rarely been conducted in so-called “modern wars” [3]—the International Rescue Committee studies in the Democratic Republic of the Congo were some of the first such surveys. It is obviously not easy to conduct large-scale population-based surveys in areas affected by conflict. But sometimes even larger challenges loom when communicating the findings. A mortality figure based on a survey may appear unambiguous, but critics tend to exploit the public's unfamiliarity with sampling methods, inferences from samples, and resulting confidence intervals to undermine the credibility of the report. Some recent publications [4,5] and a dedicated Web site [6] specifically aim to support field staff, donors, journalists, and others in understanding epidemiological studies in crisis situations.

**Linked Policy Forum**

This Perspective discusses the following new article published in *PLoS Medicine*:

Hicks MH-R, Spagat M (2008) The Dirty War Index: A public health and human rights tool for examining and monitoring armed conflict outcomes. PLoS Med 5(12): e243. doi:10.1371/journal.pmed.0050243

Madelyn Hsiao-Rei Hicks and Michael Spagat introduce the “Dirty War Index,” a public health tool that identifies rates of undesirable or prohibited war outcomes inflicted on populations during armed conflict.

## The Importance of Indices

Given the challenges in using survey data to communicate simple messages about complex phenomena, an alternative is to use indices, often in combination with league tables. A well known example is the Human Development Index (http://hdr.undp.org/en/statistics/), which is annually produced by the United Nations, based on a country's achievements in life expectancy, literacy, and gross domestic product per capita. Another example is Transparency International's Corruption Perception Index (http://www.transparency.org/policy_research/surveys_indices/cpi/), which ranks 180 countries by their perceived levels of corruption, as determined by expert assessments and opinion surveys.

In a Policy Forum in this issue of *PLoS Medicine*, Madelyn Hsiao-Rei Hicks and Michael Spagat propose a similar type of index—the Dirty War Index (DWI). The DWI aims to capture “dirty”–that is, undesirable or prohibited—war outcomes inflicted on populations during armed conflict [7]. The novelty of this index is its expression of public health findings as a ratio, in combination with a link to a specific international humanitarian law. For instance, the Geneva Conventions prohibit direct targeting of civilians. A DWI can be calculated in a specific conflict as the number of civilians killed divided by the total number of people killed. A high ratio would be telling in itself, but comparisons would also become possible, such as between different conflicts or between different war factions in the same conflict.

As the authors have started doing in their paper [7], a whole range of DWIs can be constructed, from rape to the use of prohibited weapons to the use of child soldiers, as long as acts counter to humanitarian law can be counted. The authors hope that the ease of use and understanding of DWIs will facilitate communication on the effects of war, with the ultimate goal being to moderate these effects, a similar aim to that of humanitarian law.

## Next Steps

Hicks and Spagat lay out the concept of a DWI clearly in their paper. The next step will be to apply it to specific contexts. There will be many challenges ahead, ranging from availability of high-quality and neutral data, in particular from areas where humanitarian law may be most violated, to acceptability of the DWI. Practice will show if DWIs can facilitate communication, and if they can withstand criticism from those who receive “poor” DWIs ratings.

The development of the DWI provides a good example of an answer to a recent call for better use of quantitative public health data by conflict analysts and human rights monitors [8]. As Thoms and Ron argue, human rights advocacy may benefit from enhanced collaboration between researchers applying different methodologies [8].

## Abbreviations

DWI: Dirty War Index
